# Cyclopedia of the Practice of Medicine

**Published:** 1874-12-15

**Authors:** 


CYCLOPEDIA OF THE PRACTICE OF MEDICINE. Edited by H. Von Ziemssen, Prof. of Clinical Medicine, Munich, Bavaria. Vol. 1, Acute Infectious Diseases, by Prof. Liebermeister, of Tubingen, Prof. Lebert, of Breslau, Dr. Haenisch, of Griefswald, Prof. Heubner, of Leipzig, and Dr. Oertel, of Munich. Translated by R. H. Fitz, M.D., and Chas. P. Putnam, M.D., of Boston; Arthur Von Harlinger, M.D., of Philadelphia; James T. Whittaker, M.D., of Cincinnati; Edward W. Schauffler, M.D., of Kansas City; and Francis Delafield, M.D., Horatio Bridge, M.D., Thos. E. Satterthwaite, M.D., Lewis A. Stinson, M.D., J. Haven Emerson, M.D., and Normand Smith, M.D. of New York. Albert H. Buck, M.D., New York, Editor of American Edition. New York: Wm. Wood & Co., 27 Great Jones Street. 1874.

The above is the title page to the first volume of a new Cyclopedia of Practical Medicine, to be completed in a series of similar volumes. It is a volume of 700 large octavo pages, on good paper, plain type, and a good style of cloth binding. The publishers of this American edition have done their part of the work in a highly creditable manner.

So far as we can judge the translators have also performed their respective tasks well. The subjects embraced in this volume are: Typhoid Fever, by Liebermeister; Relapsing Fever, Typhus Fever and Cholera, by Lebert; the Plague, by Liebermeister; Yellow Fever, by Haenisch; Dysentery, by Heubner; Epidemic Diphtheria, by Oertel. These subjects are treated fully and ably, as would be expected from the high reputation of the writers. And to all practitioners who desire a work of reference that will give them a reliable view of the present status of Special Pathology and Therapeutics in Europe, we freely commend this work, so far as the present volume may be regarded as representative of those that are to follow.

It is not adapted for use as a text-book for students, neither could we commend unreservedly all the views advanced in reference to the treatment of the several diseases above named.

The statement of Dr. Liebermeister that the great object of treatment in Typhoid Fever is to reduce the temperature, and that it makes but little difference what means are chosen for that purpose, provided they are efficient, appears to us indicative of a very narrow view of the essential pathology of the disease, as well as too little regard for differences in the modus operandi of medicines. Neither will our personal observation quite justify the idea that from scruple to drachm doses of quinine, even when repeated only every second day, are entirely safe in all cases of typhoid fever. It is no part of our present purpose, however, to criticise the views of any of the writers in the volume before us, but simply to announce its publication and strongly commend it to such of our readers as desire a more complete work for reference than is afforded by any of the individual works on practical medicine accessible to them.

